# Quantitatively Exploring Giant Optical Anisotropy of Quasi-One-Dimensional Ta_2_NiS_5_

**DOI:** 10.3390/nano13243098

**Published:** 2023-12-07

**Authors:** Qihang Zhang, Honggang Gu, Zhengfeng Guo, Ke Ding, Shiyuan Liu

**Affiliations:** 1 State Key Laboratory of Intelligent Manufacturing and Technology, Huazhong University of Science and Technology, Wuhan 430074, China; qihangzhang@hust.edu.cn (Q.Z.); zhengfengguo@hust.edu.cn (Z.G.); shyliu@hust.edu.cn (S.L.); 2 Guangdong Provincial Key Laboratory of Manufacturing Equipment Digitization, Guangdong HUST Industrial Technology Research Institute, Dongguan 523003, China; 3 Optics Valley Laboratory, Wuhan 430074, China; 4 Wuhan China Star Optoelectronics Semiconductor Display Technology Co., Ltd., Wuhan 430078, China; dingke1@tcl.com

**Keywords:** Ta_2_NiS_5_, optical anisotropy, Mueller matrix spectroscopic ellipsometry, dielectric tensor, critical points analysis

## Abstract

Optical anisotropy offers a heightened degree of flexibility in shaping optical properties and designing cutting-edge devices. Quasi-one-dimensional Ta_2_NiS_5_, with giant optical anisotropy, has been used in the development of new lasers and sensors. In this research endeavor, we successfully acquired the complete dielectric tensor of Ta_2_NiS_5,_ utilizing the advanced technique of Mueller matrix spectroscopic ellipsometry, enabling a rigorous quantitative assessment of its optical anisotropy. The results indicate that Ta_2_NiS_5_ demonstrates giant birefringence and dichroism, with Δ*n_max_* = 1.54 and Δ*k_max_* = 1.80. This pursuit also delves into the fundamental underpinnings of this optical anisotropy, drawing upon a fusion of first-principles calculations and critical points analysis. The anisotropy of Ta_2_NiS_5_ arises from differences in optical transitions in different directions and is shown to be due to van Hove singularities without exciton effects. Its giant optical anisotropy is expected to be useful in the design of novel optical devices, and the revelation of the physical mechanism facilitates the modulation of its optical properties.

## 1. Introduction

Two-dimensional (2D) or quasi-one-dimensional (quasi-1D) materials with in-plane anisotropy, such as PdSe_2_ [1], ReS_2_ and ReSe_2_ [2,3], ZrTe_5_ [4], CrPS_4_ [5], TiS_3_ and ZrS_3_ [6], have been hot topics in research since the study of black phosphorus (BP) [7,8,9]. These types of materials not only exhibit richer physical properties, but also provide an additional degree of freedom to modulate physical properties [10], leading to the design of novel electronic, optical, thermal, and optoelectronic devices, a feature that is not available in isotropic materials. In recent years, the exploration of new applications for low-dimensional materials has triggered extensive research into their optical anisotropy [11,12]. However, it is worth noting that most of the studies have focused on the qualitative observation and exploration of optical anisotropy phenomena, as well as designing devices based on characterization and experience. Quantitative measurements of optical anisotropy and the intrinsic formation mechanisms have not received significant attention.

The optical anisotropy of low-dimensional materials is usually described quantitatively using the dielectric tensor [13,14]. The different elements of the dielectric tensor provide quantitative information about the response of a material to electric fields in different directions, which helps to understand and predict the optical behavior of anisotropic materials for the design and optimization of optical and electronic devices. Therefore, the measurement of the complete dielectric tensor is of great significance in anisotropic materials research and applications. In addition, the physical formation mechanism of the characteristic spectral absorption structures of the dielectric function spectrum needs to be explored urgently, which will help to analyze and explain the mechanism of optical anisotropy.

Currently, techniques for determining the dielectric tensor of anisotropic materials include reflection-based methods [15], scattering-type scanning near-field optical microscopy (s-SNOM) based on near-field imaging [16,17,18], and polarization-based ellipsometry [19,20,21]. Conventional reflection-based methods generally need to be combined with the Kramers-Kronig (K-K) relations to obtain the dielectric tensor [22,23]. This method leads to inaccurate dielectric tensor measurements due to the fact that the wavelength measurement range of the instrument cannot reach infinity, and the K-K relations need to be approximated [24]. s-SNOM can accurately obtain the dielectric tensor of uniaxial crystals, but measurements of biaxial crystals need to be made with the help of other techniques to determine the position of the in-plane principal axes first [16]. Mueller matrix spectroscopic ellipsometry (MMSE) [25,26,27], on the other hand, can obtain the dielectric tensor accurately and completely and does not require a priori knowledge of thickness or crystal axis orientation [28,29,30]. A systematic approach based on first-principles calculations and critical points analysis [31] can be used to reveal the correlation between the absorption peaks of the dielectric function and the optical transitions [4,20,32], including the center energy of the transitions, their positions in the Brillouin zone (BZ), the energy bands involved in the transitions, and the types of carriers; additionally, the optical transitions analysis along different directions can reveal the physical origin of the optical anisotropy.

The quasi-one-dimensional dimetallic sulfide Ta_2_NiS_5_ has attracted much attention in recent years as an emerging low-dimensional material [33,34,35]. Ta_2_NiS_5_ has great potential as a broadband saturable absorber [36] for applications, such as infrared lasers [37,38,39,40], sensors [41], and photodetectors [42]. Its electrical and thermal anisotropy have been reported successively [43,44], and it exhibits a giant optical anisotropy; thus it may have promising prospects for applications in polarization devices, nonlinear optics, and sensors.

In this work, we use MMSE to accurately obtain the complete dielectric tensor of quasi-one-dimensional Ta_2_NiS_5_ over a wide spectral range of the UV-Vis-NIR (193 nm–1690 nm) and combine it with first-principles calculations and critical points analyses to quantitatively study its giant optical anisotropy in depth. Raman spectroscopy is used to characterize the structural information of the Ta_2_NiS_5_ sample. Angle-Resolved Raman Spectroscopy, polarization-resolved reflectance spectroscopy, and azimuthally resolved MMSE spectroscopy measurements qualitatively demonstrate its optical anisotropy, and the full permittivity tensor of Ta_2_NiS_5_ is obtained by constructing a simulation of the combined MMSE spectra. The critical points analysis of the absorption transitions corresponding to the characteristic peaks of the dielectric function and the energy band structure and the density of joint states of Ta_2_NiS_5_ are calculated by combining with the first nature principle, which reveals the physical formation mechanism of the optical anisotropy.

## 2. Materials and Methods

### 2.1. Preparation and Characterization of Ta_2_NiS_5_ Single Crystals

High-quality Ta_2_NiS_5_ single crystals were purchased from Shanghai Onway Technology Co., Ltd., Shanghai, China (link: onway-tec.com accessed on 1 October 2023) These samples are prepared using the chemical vapor transport (CVT) method from high-purity raw materials and can be up to millimeters in size. The LabRAM HR800 (HORIBA Jobin Yvon, Paris, France; link: horiba.com accessed on 2 November 2023) laser confocal Raman spectrometer was used to characterize the quasi-one-dimensional structure of Ta_2_NiS_5_ and observe its optical anisotropy phenomena. A micro-UV-visible near-infrared spectrophotometer (Jasco MSV-5200, JASCO Corporation, Tokyo, Japan; link: jascoinc.com accessed on 2 November 2023) and a commercial Muller matrix spectroscopic ellipsometer (MMSE, ME-L Mueller matrix spectroscopic ellipsometry, Wuhan E-optics Technology Co., Ltd., Wuhan, China; link: eoptics.com.cn accessed on 14 July 2023) were also employed to qualitatively characterize the anisotropy of Ta_2_NiS_5_. The MMSE was also used to obtain the Muller matrix spectra at room temperature with its 200 μm probing spot and ulteriorly acquire the complete dielectric tensor. In addition, the CCD camera on the MMSE helps to determine the measurement position on the sample at the same point.

### 2.2. First-Principles Calculations

All calculations were performed with the Vienna Ab initio Simulation Package (VASP v5.4.4). The Perdew–Burke–Ernzerhof (PBE) functional of generalized gradient approximation (GGA) based on the projected augmented wave (PAW) pseudopotentials was used to describe the exchange correlation potential. In the optimization of geometric structure, we used the PBE functional based on PAW pseudopotential, with an energy cutoff of 400 eV and an 8 × 8 × 2 Г-centered k-point mesh. When calculating the projected density of states (PDOS), a denser 16 × 16 × 4 Г-centered k-point mesh was used. The convergence criterion of force is 0.01 eV/Å and of total energy is 10^−5^ eV. The effect of spin–orbit coupling (SOC) was considered in all calculations.

## 3. Results and Discussion

### 3.1. Quasi-One-Dimensional Structure and Optical Anisotropy of Ta_2_NiS_5_

**Figure 1:**
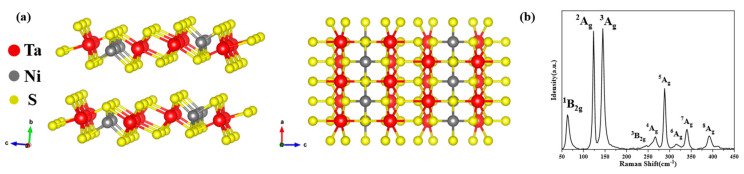
Quasi-one-dimensional structure of Ta_2_NiS_5_: (**a**) Lattice structure of Ta_2_NiS_5_, side view on the left, top view on the right; (**b**) Raman spectra of bulk Ta_2_NiS_5_.

Ta_2_NiS_5_ is a ternary chalcogenide compound with a typical van der Waals layered structure, belonging to the orthorhombic system (*Cmcm* space group, lattice constant a = 3.415 Å, b = 12.146 Å, c = 15.097 Å) at room temperature [33,35,45]. Figure 1a shows its quasi-one-dimensional structure. In the a-c plane, Ni and Ta atoms are combined with S atoms to form a Ni-S_4_ tetrahedron and Ta-S_6_ octahedron, respectively. Ni-S_4_ tetrahedrons and Ta-S_6_ octahedrons are each combined into linear chains along the a-axis and arranged into planes in the manner of two Ta-S_6_ octahedron chains per one Ni-S_4_ tetrahedron chain along the c-axis [35]. This different atomic arrangement of Ta_2_NiS_5_ along the a-axis and c-axis also predicts its giant in-plane optical anisotropy. The experimental bulk sample is long and oriented along the long strip in the a-axis, which is related to its chain structure along the a-axis, which we verified later through the results of first-principles calculations. Figure 1b shows the Raman spectrum of the bulk Ta_2_NiS_5_, showing 7 A_g_ modes (^2^A_g_: 123.6 cm^−1^, ^3^A_g_: 145.1 cm^−1^, ^4^A_g_: 266.3 cm^−1^, ^5^A_g_: 288.7 cm^−1^, ^6^A_g_: 317.2 cm^−1^, ^7^A_g_: 340.1 cm^−1^, ^8^A_g_: 392.6 cm^−1^) and 2 B_2g_ modes (^1^B_2g_: 63.8 cm^−1^, ^3^B_2g_: 262.1 cm^−1^). Previous studies have shown that Ta_2_NiS_5_ has 8 A_g_ modes and 3 B_2g_ modes [33,46], which matches the measurements in Figure 1b, confirming the structure of the bulk sample. It should be noted that the ^1^A_g_ mode is less than 50 cm^−1^, which is beyond the measurement range of the instrument, and the ^2^B_2g_ mode is between ^2^A_g_ and ^3^A_g_, which cannot be visualized due to the small intensity of ^2^B_2g_ and the high intensity of ^2^A_g_ and ^3^A_g_.

**Figure 2:**
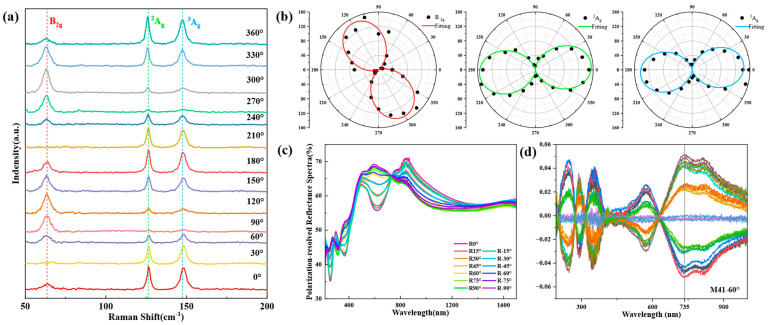
Qualitative characterization of the giant optical anisotropy of Ta_2_NiS_5_: (**a**) polarized Raman spectra of Ta_2_NiS_5_; (**b**) polar plot and fitting of B_2g_, ^2^A_g_ and ^3^A_g_ Raman modes; (**c**) reflectance spectra at different polarization angles of Ta_2_NiS_5_; (**d**) experimental spectra at different azimuth angles at an incident angle of 60° of Muller matrix elements M41, which is one of the off-diagonal elements in the fourth row and the first column of the 4 × 4 Mueller matrix.

The optical anisotropy of Ta_2_NiS_5_ was observed using angle-resolved Raman spectroscopy, as shown in Figure 2a (three Raman modes ^1^B_2g_ [in other words, the following B_2g_], ^2^A_g_, and ^3^A_g_, were chosen here as representatives, and their vibrational modes are shown in Figure S1). With the change in Raman laser polarization direction, the angle between the laser electric field direction and the crystal optical axis direction is changed, resulting in a periodic change in the Raman scattered light intensity for all of the different modes, of which the polar plots of the light intensity for the B_2g_, ^2^A_g_ and ^3^A_g_ three vibrational modes are shown in Figure 2b. We theoretically calculated the Raman intensity [47] versus angle change (see Supplementary Materials), which showed that the curve in Figure 2b and the theoretical calculation results are in good agreement with the experimental results.

In addition, we also measured the reflectance of Ta_2_NiS_5_ using a micro-distinguished photometer, as shown in Figure 2c, which also showed significant differences in reflectance under the change in polarization angle from −90° to 90°, which is related to the relative positions of the optical axis direction and the polarization direction. In these data, R0° corresponds to the a-axis direction R_a_ and R90°/−90° to the c-axis direction R_c_.

MMSE is a non-contact measuring instrument widely used to characterize the thickness and optical properties of nanomaterials. It generates polarized light through the polarizing arm and detects the change in the polarized light in the detector arm after reflection from the sample, thus obtaining the properties of the material. Unlike conventional ellipsometry, it can measure the generalized ellipsometric parameters (the 15 components of the 4 × 4 normalized Mueller matrix), which can be converted into the 2 × 2 Jones matrix when the depolarization effect is neglected. It should be noted that the Mueller matrices obtained from the measurements in this work are all normalized. The off-diagonal elements of the Mueller matrix reflect the anisotropy of the material, and the spectrum of one of the off-diagonal elements of the Mueller matrix, M41, at an incident angle of 60° is shown in Figure 2d. For the experiments, we placed the a-axis of the bulk sample parallel to the x-axis of the ellipsometric coordinate system when the azimuth angle was 0°, which helped to simplify the rotation of the coordinate system during the ellipsometric analysis. The values of the off-diagonal elements of the Mueller matrix are affected by both the magnitude of the anisotropy of the material and the orientation of the optical axis of the sample during the measurement. Thus, we observed the changes in the M41 values by rotating the sample in the a-c plane to change the orientation of the optical axis; this angle of rotation is called the ellipsometric azimuth angle (Figure S5). The azimuths of the curves of the same color in Figure 2d differ by 180°, and it can be seen that the values of the element M41 at the same wavelength such as 735 nm show a clear periodicity.

### 3.2. Quantitative Characterization of the Optical Anisotropy of Ta_2_NiS_5_

The complete Mueller matrix spectrum acquired by MMSE was used to extract the dielectric tensor. This process was realized by building a reasonable optical model that includes a suitable dispersion model composed of several contributions describing individual absorption structures to match the spectrum of the Mueller matrix. The individual contributions were modeled using the Tauc–Lorentz model [48] or using the model of Gaussian broadened harmonic oscillators (a Gaussian curve for *ε*_2_, and an *ε*_1_ curve that maintains Kramers–Kronig agreement [49]). We used the root mean square error *RMSE* to quantitatively evaluate how well the fitted data matched the experimental data, defined as [4]:

(1) $$RMSE=\sqrt{\frac{1}{15L-h}\sum_{l=1}^{L}\sum_{i,j=1}^{4}{\left(M_{i,j}^{c}-M_{i,j}^{m}\right)}^{2}}\times 1000$$

where *M^c^_i_*_,*j*_ and *M^m^_i_*_,*j*_ denote the calculated and measured values of the Mueller matrix, the subscript *i*,*j* denotes the *i*th row and the *j*th column, *L*, *l* denotes the total number of points and the *l*th point of the measurement, and *h* denotes the number of fitted parameters. In general, a smaller *RMSE* indicates a better fit, and a good model should have an RMSE of less than 10.

The Ta_2_NiS_5_ sample in our experiments was bulk. It reaches a thickness of several hundred micrometers, in which the dielectric shielding effect is weak, and there is no need to consider the effect of thickness on optical properties. Therefore, the optical model was built by treating it directly as a substrate to analyze; in other words, only the optical constants of the sample (and not the thickness of the sample) were fitted. The fitting results show *RMSE* = 7.795 (more details in Supplementary Materials), which indicates that the optical constants obtained have a fairly high accuracy and verifies our rationale for analyzing the bulk sample as a substrate.

For an orthorhombic crystal such as Ta_2_NiS_5_, the dielectric tensor is a 3rd-order diagonal matrix, with the main diagonal elements being the dielectric function in the direction of each of the three crystal axes, and containing one real and one imaginary part each:

(2) $$\varepsilon =\left[\begin{array}{ccc} \varepsilon_{a} & & \\ & \varepsilon_{b} & \\ & & \varepsilon_{c} \end{array}\right]=\left[\begin{array}{ccc} \varepsilon_{r,a}-i\varepsilon_{i,a} & & \\ & \varepsilon_{r,b}-i\varepsilon_{i,b} & \\ & & \varepsilon_{r,c}-i\varepsilon_{i,c} \end{array}\right]$$

**Figure 3:**
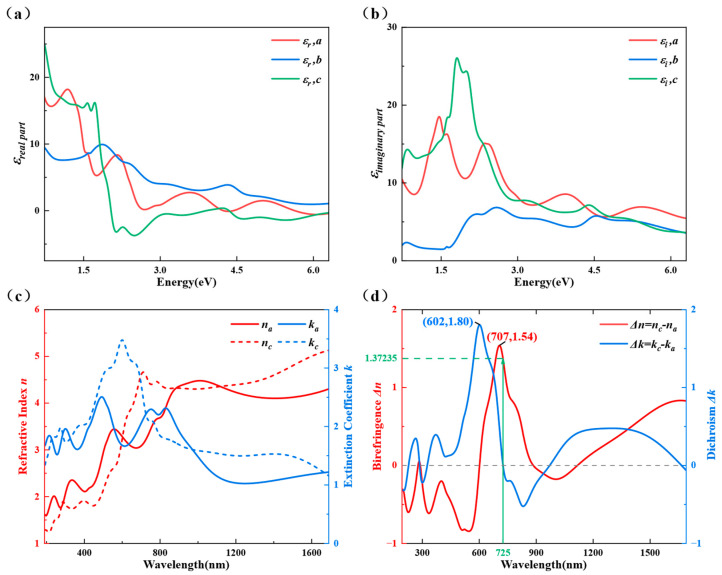
The complete dielectric tensor and in-plane (the (**a**–**c**) plane) optical constants of Ta_2_NiS_5_: (**a**) real part of the dielectric tensor; (**b**) imaginary part of the dielectric tensor; (**c**) in-plane refractive index (*n)* and extinction coefficient (*k*; the complex refractive index); (**d**) birefringence (Δ*n)* and dichroism (Δ*k)*.

Figure 3a,b show the real and imaginary parts of the dielectric function of Ta_2_NiS_5_ obtained by fitting the Mueller matrix. There is a large difference between the dielectric functions of the three crystal axes. The difference in the b-axis, which is bonded by van der Waals forces along the interlayer direction, is particularly obvious compared to the a- and c-axes, whereas the a-c plane is bonded by the covalent bonds of Ta-S and Ni-S. The differences between a- and c-axes are thought to be due to the different atomic arrangements resulting from quasi-one-dimensional chain structures, causing different quantities and rows of the two kinds of covalent bonds in the two directions. In the complex refractive index, *N_x_* = *n_x_* − *ik_x_* = $\sqrt{\varepsilon_{x}}$, where the subscript *x* denotes the three crystal axes of the orthorhombic crystal system *a*, *b* and *c*, *n* is the refractive index and *k* is the extinction coefficient. The complex refractive index tensor is calculated from the dielectric tensor, as shown in Figure 3c. The in-plane anisotropy is expected to be used in the design of new devices, so only the complex refractive indices for the a-axis and c-axis are given here. The *n* and *k* in both directions are very different in both their waveform and amplitude, quantitatively showing the giant optical anisotropy of Ta_2_NiS_5_.

The in-plane anisotropy is often quantitatively described by two parameters: birefringence Δ*n = n_c_* − *n_a_* and dichroism Δ*k = k_c_* − *k_a_* [50,51]. These parameters can be calculated from the previously obtained in-plane complex refractive indices, as shown in Figure 3d. The absolute values of birefringence and dichroism are mostly higher than 0.2 in the measured wavelength range and even exceed 1 in some wavelength ranges, which is a good indication that its optical anisotropy is giant. In the measured wavelength range, there is maximum birefringence Δ*n_max_* = 1.54 at a wavelength of 707 nm and maximum dichroism Δ*k_max_* = 1.80 at a wavelength of 602 nm. And at a wavelength of 725 nm, the birefringence Δ*n* = 1.37 and the dichroism Δ*k* = 0 are expected to be used for designing waveplates. Compared with some other low-dimensional materials and waveplate materials, such as BP [52] and rutile [53], its giant optical anisotropy has an obvious advantage, and it has great potential in realizing the miniaturization and integration of optical devices.

The complex refractive index can also be used to calculate the reflectance of orthorhombic crystal systems such as Ta_2_NiS_5_ [13]:

(3) $$R_{m}=\frac{{\left(n_{m}-1\right)}^{2}+k_{m}{}^{2}}{{\left(n_{m}+1\right)}^{2}+k_{m}{}^{2}}$$

where the subscript *m* denotes the three crystal axes of the orthorhombic crystal system *a*, *b*, *c*, and *R*, *n*, and *k* represent the reflectance, refractive index, and extinction coefficient in this direction, respectively. After obtaining the complex refractive index, its reflectance was calculated using Equation (3) and verified by experimental measurements (Figure S5). The calculated reflectance matches well with the actual measured reflectance, especially at the peak and valley positions. Even some inconspicuous peaks are reflected in the theoretically calculated reflectance. This proves the accuracy and reliability of our complex refractive index obtained by MMSE from the side. In addition, the optical constants obtained from the first nature principles calculation of the optical properties of Ta_2_NiS_5_ are also in high agreement with those obtained experimentally, (Figure S6), which, likewise, validates our experimental results.

### 3.3. Critical Points and Optical Transitions

**Figure 4:**
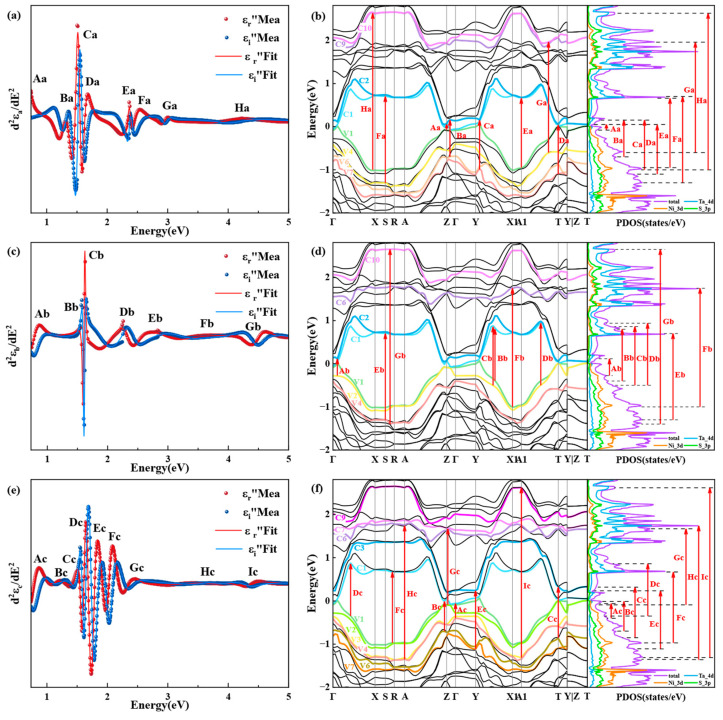
The critical points (CPs) and their physical origin were analyzed along the three axes of the Ta_2_NiS_5_ crystal, combined with the band structure and projected density of states (PDOS). The second derivative of the dielectric functions was calculated with respect to energy along the a-axis (**a**), b-axis (**c**), c-axis (**e**) for CP analysis. The corresponding interband transition of CP points along the a-axis (**b**), b-axis (**d**), c-axis (**f**) in the band structure (left) and PDOS (right) of Ta_2_NiS_5_ is shown. A_(a,b,c)_ to I_(a,b,c)_ indicate the CP points position from low to high central energy, and the subscript indicates the axial direction. V*p* and C*q* represent the *p*th highest valence band and the *q*th lowest conduction band, respectively.

In order to better comprehend the giant optical anisotropy of Ta_2_NiS_5_, we performed critical points (CP) and optical transitions analysis of its dielectric function in the three crystal axis directions. Critical points analysis is performed by varying different parameters of the critical points to fit the second-order partial derivatives of the dielectric function with respect to energy [31]:

(4) $$\frac{d^{2}\varepsilon }{dE^{2}}=\{\begin{array}{c} n(n-1)Ae^{i\phi }{\left(E-E_{0}+i\Gamma \right)}^{n-2}(n\neq 0)\\ Ae^{i\phi }{\left(E-E_{0}+i\Gamma \right)}^{-2}(n=0) \end{array}$$

where *A*, *ϕ*, *E*_0_, and Г denote the amplitude, phase, center energy, and damping coefficient of the critical points, respectively, *i* is an imaginary unit, and *n* denotes the dimensions of the optical transitions involved in the critical points (including three-dimensional, two-dimensional, one-dimensional, and zero-dimensional), and the corresponding *n* is 1/2, 0, −1/2, and −1, respectively. More details about CP analysis can be found in the Supplementary Materials. Figure 4a,c,e show the second-order derivatives of the nodal functions with respect to the energy *d*^2^*ε/dE*^2^ and their best-fit curves for each of the three directions. There are 9, 9, and 11 0D critical points along the a-axis, b-axis, and c-axis directions in the measured spectral region, from which the center energy of the corresponding optical transitions is determined, and the parameters related to the CP points are detailed in Table S2. The energy band structure and PDOS obtained from first-principles calculations can be linked to the CP points. As shown in Figure 4b,d,f, the center energy value of the CP points is used to identify where its optical transitions occur in the energy band structure and to recognize its carrier type in the PDOS.

For Ta_2_NiS_5_, there is a large no-band range between the twelfth and thirteenth low guide bands, and electrons in lower valence bands are more difficult to excite; thus, only optical transitions below 4.5 eV are considered. In Figure 4b, the critical point A_a_ with a center energy *E*_0_ of 0.53 eV (in other words, *E*_c-v_ = *E*_0_ = 0.53 eV) represents the optical transition from V1 to C1 located at the high-symmetry point Z in the Brillouin zone. The critical point B_a_ (*E*_0_ = 1.25 eV) indicates the optical transition from V6 to C2 between points Z and Γ in the Brillouin zone. The critical point C_a_ (*E*_0_ = 1.53 eV) appears in k-space between the points Y and X1, corresponding to the optical transition from V7 to C2. There are three critical points D_a_ (*E*_0_ = 1.55 eV), *E*_a_ (*E*_0_ = 2.38 eV), and F_a_ (*E*_0_ = 2.40 eV) at the high-symmetry points T, A1, and S in the Brillouin zone, corresponding to the optical transitions from V7 to C2, from V1 to C2, and from V4 to C1, respectively. The critical point G_a_ (*E*_0_ = 2.97 eV) denotes the transition from V4 to C10 located between the points T and A1, and H_a_ (*E*_0_ = 4.05 eV) denotes the transition from V1 to C9 located between points Γ and X. Similarly, we can obtain the results of the optical transitions analysis for the b and c axes, as shown in Table 1.

The electronic transitions corresponding to the critical points in the three crystal axis directions show significant differences, in terms of both k-space position and energy bands involved, which is the physical explanation for the generation of the anisotropy of the dielectric function. In particular, the a-axis and c-axis directions have critical points at two identical locations in k-space at points A1 and T, suggesting that electrons here are involved in dielectric activity in both directions. In addition, the best fit of the 0D critical points illustrates the deeply localized character of the Ta_2_NiS_5_ electron wavefunction [54]. In other words, the optical transitions are dominated by multiple van Hove singularities rather than delocalized excitons [55], which matches previous reports [43,54]. Additionally, all critical points are caused by electrons in the valence band consisting of hybridized Ni-3d and S-3p orbitals transitioning as main carriers to the conduction band formed by the Ta-5d orbital.

## 4. Conclusions

In summary, this investigation quantitatively analyzes the complete dielectric tensor of quasi-one-dimensional Ta_2_NiS_5_ in the UV-Vis-NIR spectral range, examines its giant optical anisotropy, and probes the physical origin of the anisotropy in conjunction with CP analysis and first-principles calculations. Angle-resolved Raman spectroscopy, polarization-resolved reflectance spectroscopy, and azimuthally resolved Mueller matrix spectroscopy qualitatively observe the optical anisotropy phenomenon in Ta_2_NiS_5_. The dielectric tensor of Ta_2_NiS_5_ over a wide spectral range is determined by MMSE, and accurate numerical curves of its birefringence and dichroism are obtained, which provide data support for possible device design, and its giant optical anisotropy is of great advantage and prospect in realizing the miniaturization and integration of polarized optics devices. The CP analysis and the optical transitions analysis show, from a quantum mechanical point of view, that the fundamental reason for the differences in the properties of the three crystal axis orientations of Ta_2_NiS_5_ is the differences in the optical transitions corresponding to the CPs. It is also shown that Ta_2_NiS_5_ does not exhibit delocalized exciton transitions but rather has multiple van Hove singularities. Our work provides data and physical mechanisms for the regulation of optical properties of Ta_2_NiS_5_, enriches the database of optical constants of materials, and facilitates the further study and application of quasi-one-dimensional Ta_2_NiS_5_.

## Figures and Tables

**Table 1:** Results of optical transitions analyses in the a-, b- and c-axis directions.

Axis	Critical Point	Center Energy *E*_0_ (eV)	Position in the BZ	Energy Bands Involved in the Transition
a-axis	A_a_	0.53	Z	V1-C1
	B_a_	1.25	Z-Γ	V6-C2
	C_a_	1.53	Y-X1	V7-C2
	D_a_	1.55	T	V7-C2
	E_a_	2.38	A1	V1-C2
	F_a_	2.40	S	V4-C1
	G_a_	2.97	T-A1	V4-C10
	H_a_	4.05	Γ-X	V1-C9
b-axis	A_b_	0.77	Γ-X	V2-C2
	B_b_	1.61	Y-X1	V1-C1
	C_b_	1.78	Y-X1	V2-C1
	D_b_	1.85	A1-T	V2-C1
	E_b_	2.40	S	V4-C1
	F_b_	3.15	X1	V1-C6
	G_b_	4.44	S-R	V4-C10
c-axis	A_c_	0.75	Γ	V4-C1
	B_c_	1.08	A-Γ	V6-C1
	C_c_	1.59	T	V6-C3
	D_c_	1.61	Γ-X	V1-C1
	E_c_	1.76	Y	V7-C3
	F_c_	2.04	S-R	V1-C1
	G_c_	2.32	Z-Γ	V2-C7
	H_c_	3.49	A	V4-C6
	I_c_	3.80	A1	V3-C9

## Data Availability

The data presented in this study are available on request from the corresponding author.

## Supplementary Materials

The following supporting information can be downloaded at: https://www.mdpi.com/article/10.3390/nano13243098/s1, Figure S1. Schematic illustration of atomic displacements in the three vibration modes. Figure S2. Schematic diagram of Raman measurement experiment. Figure S3a. Schematic diagram of Mueller matrix ellipsometry principle. Figure S3b. Schematic diagram of an ellipsometry experiment. Figure S4. Mueller matrix spectra of Ta_2_NiS_5_ with incidence angles from 55° to 70° and their best-fit curves. Figure S5. Experimental and calculated reflectance spectra of a-axis and c-axis. Figure S6. Optical constants calculated from first principles. Table S1. Best-fit parameters for physical oscillators in anisotropic ellipsometric fitting. Table S2. Best-fit parameters for Ta_2_NiS_5_ critical points analysis. Refs. [4,33,46,47] are cited in the Supplementary Materials.
