# Supplementary material for: Quantitatively Exploring Giant Optical Anisotropy of Quasi-One-Dimensional Ta2NiS5
Source: Nanomaterials (Basel). 2023 Dec 7;13(24):3098. doi: 10.3390/nano13243098 (PMC10745865; doi:10.3390/nano13243098)
Supplement: Supplementary file 1 [file nanomaterials-13-03098-s001.zip › nanomaterials-2728618-supplementary.pdf]

Supplementary Materials:

Quantitatively exploring giant optical anisotropy of quasi-one-dimensional Ta_2NiS_5

Qihang Zhang [1], Honggang Gu [1,2,4*], Zhengfeng Guo [1], Ke Ding [3], Shiyuan Liu [1,4]

[1] State Key Laboratory of Intelligent Manufacturing and Technology, Huazhong University of Science and Technology, Wuhan 430074, P. R. China;
[2] Guangdong HUST Industrial Technology Research Institute, Guangdong Provincial Key Laboratory of Manufacturing Equipment Digitization, Dongguan, Guangdong 523003, China
[3] Wuhan China Star Optoelectronics Semiconductor Display Technology Co., Ltd., Wuhan, Hubei 430078, China;
[4] Optics Valley Laboratory, Hubei 430074, China
* Correspondence: hongganggu@hust.edu.cn

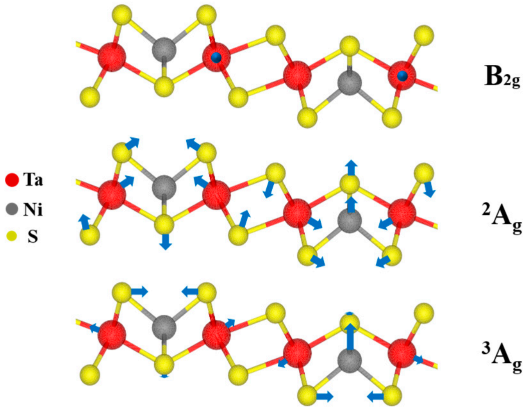

Figure S1. Schematic illustration of atomic displacements in the three vibration modes, with the view along the a-axis shown [46].

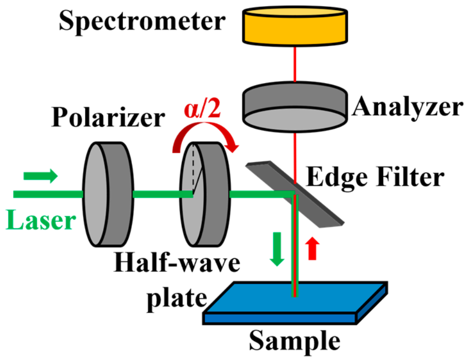

Figure S2. Schematic diagram of Raman measurement experiment. A polarized laser passes through a half-waveplate and excites the sample, and the excitation signal is detected by an analyzer placed in parallel. α is the angle between the laser polarization direction and the sample optical axis direction.

The intensity of the Raman vibrational modes is indicated by [47]:

$$I \propto \left| \boldsymbol{e}_i \boldsymbol{R} \boldsymbol{e}_o \right|^2 \qquad \text{(S1)}$$

where $\boldsymbol{e}_i$ and $\boldsymbol{e}_o$ are the unit polarization vectors of the incident and scattered laser, the incident laser polarization direction is parallel to the z-axis, and the analyzer is fixed along the z-axis, so there are $\boldsymbol{e}_i = (0\ 0\ 1)^T$, $\boldsymbol{e}_o = (0\ 0\ 1)$. $\boldsymbol{R}$ is the Raman tensor for the different modes, denoted as:

$$R_{A_g} = \begin{pmatrix} A & 0 & 0 \\ 0 & B & 0 \\ 0 & 0 & C \end{pmatrix} \quad \text{(S2a)} \qquad R_{B_{2g}} = \begin{pmatrix} 0 & 0 & E \\ 0 & 0 & 0 \\ E & 0 & 0 \end{pmatrix} \quad \text{(S2b)}$$

Define the angle between the x-axis and the a-crystal axis as β. The rotation matrix of the Raman tensor is $\boldsymbol{M}$, and the rotated Raman tensor is $\boldsymbol{R}'$,

$$\boldsymbol{M} = \begin{pmatrix} \cos\beta & 0 & -\sin\beta \\ 0 & 1 & 0 \\ \sin\beta & 0 & \cos\beta \end{pmatrix} \quad \text{(S3)}$$

$$\boldsymbol{R}' = \boldsymbol{MRM}^T \quad \text{(S4)}$$

Also rotating the half-wave plate counterclockwise requires the introduction of the Jones matrix $\boldsymbol{J}_{\lambda/2}$:

$$\boldsymbol{J}_{\frac{\lambda}{2}} = \begin{pmatrix} -\cos\alpha & 0 & -\sin\alpha \\ 0 & 0 & 0 \\ -\sin\alpha & 0 & \cos\alpha \end{pmatrix} \quad \text{(S5)}$$

Thus, the intensity of the Raman mode is described as:

$$I \propto \left| \boldsymbol{e}_o \cdot \boldsymbol{R}' \cdot \boldsymbol{J}_{\frac{\lambda}{2}} \cdot \boldsymbol{e}_i \right|^2 = \left| \boldsymbol{e}_o \cdot \boldsymbol{MRM}^T \cdot \boldsymbol{J}_{\frac{\lambda}{2}} \cdot \boldsymbol{e}_i \right|^2 \quad \text{(S6)}$$

Substituting the above equation into **Eq. S6** yields:

$$I_{A_g} \propto \left| A\sin\beta\sin(\beta-\alpha) + C\cos\beta\cos(\beta-\alpha) \right|^2 \quad \text{(S7)}$$

$$I_{B_{2g}} \propto \left| E\sin(2\beta-\alpha) \right|^2 \quad \text{(S8)}$$

which are fitted the experimental ones well in **Figure 2b**.

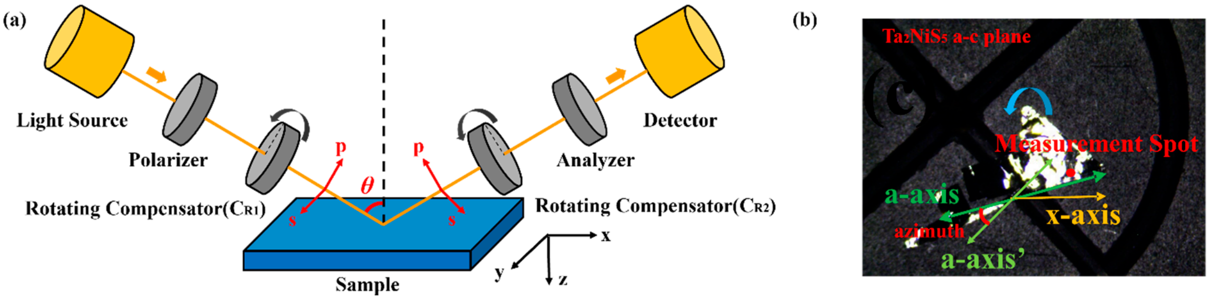

Figure S3. (a) Schematic diagram of Mueller matrix ellipsometry principle. θ denotes the angle of incidence, p and s denote the vertically decomposed components of the light wave. The coordinate system

x-y-z is the ellipsoid system coordinate system. **(b)** Schematic diagram of an ellipsometry experiment. When the sample is rotated in the a-c plane, the azimuth angle is defined as the angle between the a-axis after rotation and before rotation. For the measurement, the sample is placed on the sample stage as shown in Fig. S3b, at which time it is noted as azimuth = 0°, and then the azimuth is changed by rotating the sample stage. Measurements were made at 15-degree azimuthal intervals, and each measurement included ellipsometric data at five angles of incidence. Measurement position corresponding to the data used for ellipsometric fitting is also shown. The Euler angles ψ, θ are fitted so that the crystal axes are parallel to the elliptic-partial coordinate system, which results in ψ= -14.655°, θ= 62.127° after an anisotropic fit such that the a-axis and c-axis are parallel to the x-axis and y-axis, respectively.

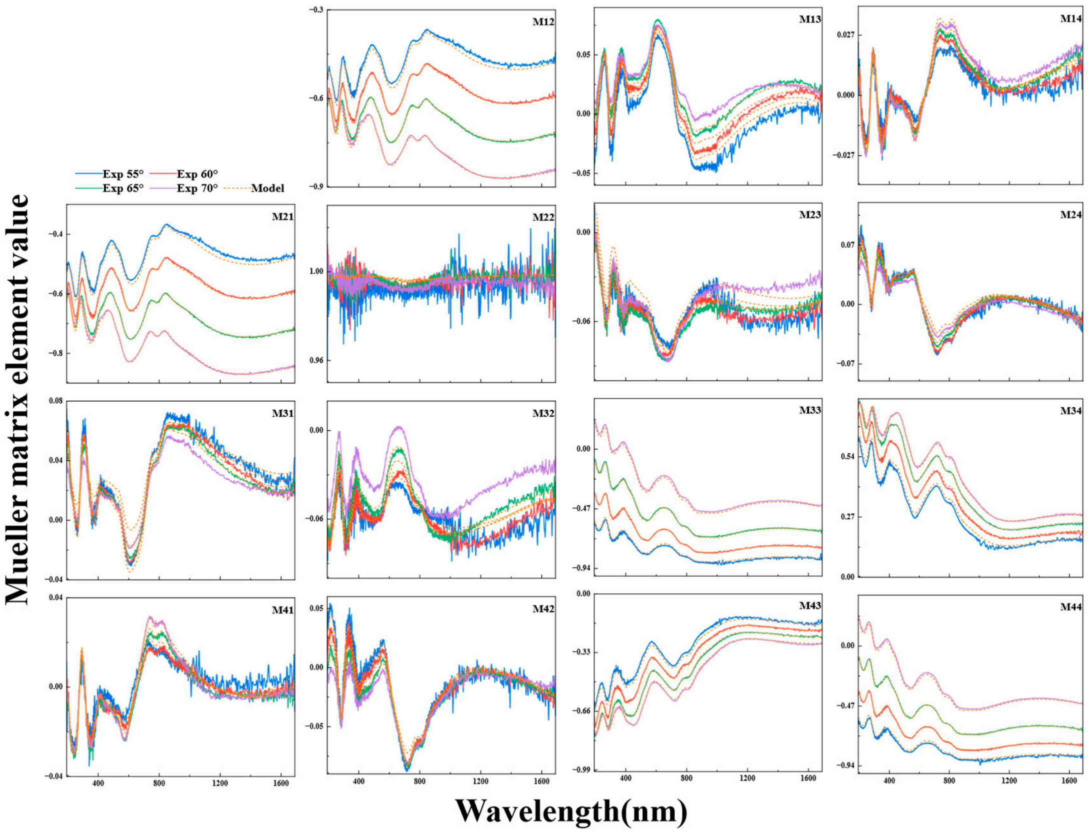

Figure S4. Mueller matrix spectra of Ta_2NiS_5 with incidence angles from 55° to 70° and their best-fit curves.

The ellipsometric data analysis is done by modeling and fitting the experimental data to get the results, where the model includes both the physical structure and the optical constants of each part. In our experiments, the physical structure consists of Ta_2NiS_5 single crystals and the air above them, where (1) the roughness is not considered, and its fitting or not has almost no effect on the RMSE; (2) since the thickness has no effect on the fitting process, the Ta_2NiS_5 bulk samples are analyzed directly as the substrate; and (3) the Ta_2NiS_5 is opaque, and therefore the effect of backside emission is not considered, and the mathematical model B-spline is directly used in the fitting. For the optical constants, the dielectric tensor of Ta_2NiS_5 is used as a parameter for fitting, and for orthorhombic crystal systems, the dielectric tensor can be expressed by the dielectric functions ε_a, ε_b and ε_c in the direction of the three crystal axes, which are replaced with physical oscillator models, including Gaussian model, Tauc-Lorentz model, and so on, after being fitted by the B-splines.

$$\varepsilon_k = \sum_{l}^{L} \varepsilon_{Gaussian}^{l}(A_l, \eta_l, E_{n,l}; E) + \sum_{m}^{M} \varepsilon_{Tauc-Lorentz}^{m}(A_m, \eta_m, E_{o,m}, E_{g,m}; E), k = a, b, c \qquad (S9)$$

where $\varepsilon_{Gaussian}$ and $\varepsilon_{Tauc\text{-}Lorentz}$ are the Gaussian model and Tauc-Lorentz model. They can be respectively expressed as :

$$\varepsilon = \varepsilon_r - i\varepsilon_i \qquad \text{(S10)}$$

$$Gaussian\begin{cases} \varepsilon_i = Ae^{-(\frac{E-E_n}{Br})^2} + Ae^{-(\frac{E+E_n}{Br})^2} \\ \varepsilon_r = \dfrac{2}{\pi} P\int_0^{\infty} \dfrac{\xi\varepsilon_i(\xi)}{\xi^2 - E^2} d\xi \end{cases} \qquad \text{(S11)}$$

$$Tauc-Lorentz\begin{cases} \varepsilon_i = \begin{cases} \dfrac{A\eta E_0 (E-E_g)^2}{(E^2 - E_0{}^2) + \eta^2 E^2} \bullet \dfrac{1}{E}, E > E_g \\ 0, E \le E_g \end{cases} \\ \varepsilon_r = \dfrac{2}{\pi} P\int_{E_g}^{\infty} \dfrac{\xi\varepsilon_i(\xi)}{\xi^2 - E^2} d\xi \end{cases} \qquad \text{(S12)}$$

In **Eq. S3** and **S4**, A, η, and E_n refer to the amplitude, the damping coefficient, and the center energy of the oscillators, respectively. E_0 is the peak transition energy and E_g is the bandgap energy. ε_r are converted from the Kramers-Kronig relation using ε_i.

For the analysis of our data, the ellipsometric parameters ψ and Δ were first fitted using B-splines, this is because there is little priori knowledge about the optical properties of Ta2NiS5, which prevents us to construct a suitable parameterized dispersion model in the first step of the ellipsometric fitting. The B-spline function is a pure mathematical model and has the advantage that it doesn't need priori knowledge about the optical transitions of the investigated material and can quickly achieve rough initial values for the dielectric functions. Then the model was converted to a biaxial crystal and the Euler angle fitting is turned on. Since this material belongs to the orthorhombic crystal system and the angle between the three crystal axes is 90°, it is not necessary to enter the crystal axis clamping angle. Then Mueller matrix is fitted with this model, and the dielectric function of the three directions is obtained by three B-splines, and then the dielectric function of the three directions is physically parameterized, respectively. After the parameterization is completed, the Mueller matrix is fitted again, and the final result is obtained. The results show that there are 1, 0, and 3 Gaussian models and 9, 9, and 10 Tauc-Lorentz models in the a, b, and c directions, respectively, and the specific fitting results are shown in **Table S1**.

Table S1. Best-fit parameters for physical oscillators in anisotropic ellipsometric fitting.

	Oscillators	Center energy(En)[eV]	Peak transition energy (E0) [eV]	Bandgap energy(Eg) [eV]	Amplitude (A)	Damping coefficient (η) [eV]
	Tauc-Lorentz	/	0.586±2.7392	0.034±2.9251	15.7966±179.81931	1.130±4.3261
	Tauc-Lorentz	/	1.487±0.0251	0.192±2.1559	5.0092±15.98085	0.312±0.0330
	Tauc-Lorentz	/	1.574±0.0088	1.508±0.0084	250.7507±81.63456	0.207±0.0168
	Tauc-Lorentz	/	2.372±0.0158	0.813±0.6036	14.1183±11.03434	0.589±0.0363
ε_a	Tauc-Lorentz	/	4.056±0.0320	0.024±1.2011	8.6395±4.93567	1.377±0.0645
	Tauc-Lorentz	/	5.052±0.0336	4.225±0.0631	217.0681±28.01467	1.779±0.0495
	Tauc-Lorentz	/	1.289±0.0265	0.265±1.1553	3.6303±8.27404	0.377±0.0572
	Tauc-Lorentz	/	2.371±0.0143	2.558±0.0151	249.3404±82.28161	0.245±0.0853
	Tauc-Lorentz	/	2.953±0.0177	0.000±8.4413	0.1327±0.75527	0.259±0.0343
	Gaussian	7.238±0.1099	/	/	3.915150±0.5904565	0.9218±0.15382
	Tauc-Lorentz	/	0.773±0.1337	0.412±0.2913	3.9482±18.66293	0.429±0.4630
	Tauc-Lorentz	/	1.547±3.8366	3.8366±6.5043	4.7297±74.42277	1.846±4.3057
	Tauc-Lorentz	/	1.612±0.0266	0.000±56.5519	0.0219±1.52982	0.063±0.0471
	Tauc-Lorentz	/	1.938±0.1079	1.565±0.1008	81.9329±51.357	0.936±0.2184
ε_b	Tauc-Lorentz	/	2.398±0.1010	2.254±0.0574	106.4267±67.57983	0.596±0.1742
	Tauc-Lorentz	/	3.150±0.2354	2.840±0.1317	73.4194±50.74651	1.107±0.2261
	Tauc-Lorentz	/	4.469±0.0678	3.534±1.1290	24.9346±58.08078	0.567±0.2045
	Tauc-Lorentz	/	5.113±0.2806	3.714±0.9289	46.4110±150.87999	1.670±1.3951
	Tauc-Lorentz	/	6.417±24.0344	4.296±8.3134	37.1567±929.44771	6.124±102.6502
	Tauc-Lorentz	/	0.769±0.1653	0.431±0.4734	29.8006±109.89458	0.456±0.2421
	Gaussian	1.205±0.3693	/	/	7.189544±19.7884447	0.4377±0.92859
	Gaussian	1.511±0.1686	/	/	9.305308±16.1119673	0.3452±0.40304
	Tauc-Lorentz	/	1.611±0.0457	1.334±0.87	35.7172±230.12087	0.169±0.1068
	Tauc-Lorentz	/	1.752±0.0383	1.548±0.1341	277.4768±393.27676	0.264±0.0756
	Tauc-Lorentz	/	2.032±0.0703	0.722±8.5309	7.9328±98.42038	0.299±0.1107
ε_c	Tauc-Lorentz	/	2.280±0.0814	1.926±0.5644	92.5269±164.28007	0.469±0.3120
	Tauc-Lorentz	/	3.101±0.1582	2.475±0.5272	53.7244±73.70178	0.960±0.3423
	Tauc-Lorentz	/	4.130±0.4930	0.000±81.1032	2.0155±80.73983	0.948±1.3786
	Tauc-Lorentz	/	4.359±0.1007	3.797±0.7138	61.2940±225.80516	0.502±0.2897
	Tauc-Lorentz	/	5.099±0.2584	3.677±2.2319	39.8543±86.51341	1.332±0.7867
	Tauc-Lorentz	/	6.201±0.3838	5.203±1.7988	38.7830±105.46711	1.132±0.5109
	Gaussian	0.006±960.61	/	/	793.496577±0.103	0.0073±240.88

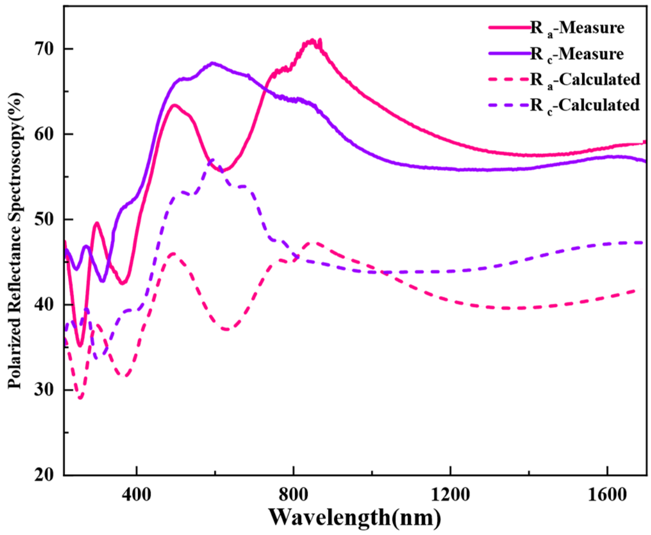

Figure S5. Experimental and calculated reflectance spectra of a-axis and c-axis. The difference in values is mainly due to that the measured ellipsometric data may fluctuate greatly depending on the measurement environment, the signal-to-noise ratio of the beam, the sample, the incidence and so on, but the positions of the featured absorbing peaks reflected by the measured ellipsometric spectra remain unchanged with these factors. Therefore, the absolute values of the optical constants extracted by the mathematical fitting from the measured ellipsometric data may deviate from their real values and notably differ from each other in different measurement conditions, which ultimately leads to a discrepancy between the calculated reflectance value using the extracted optical constants and the measured one by the spectrophotometer.

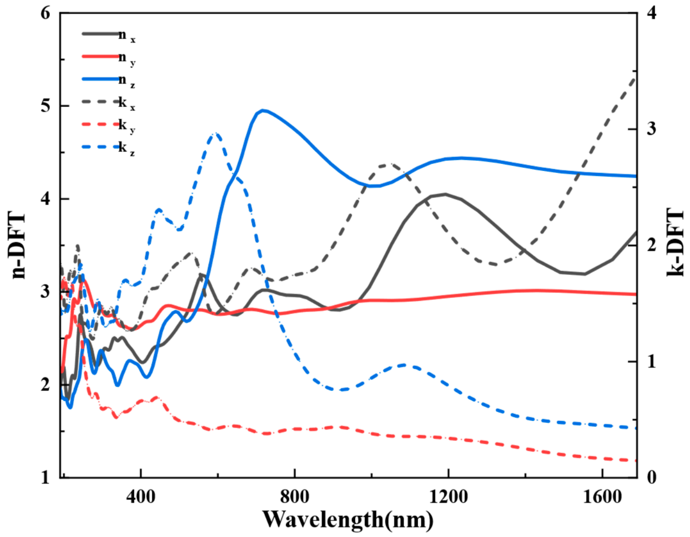

Figure S6. Optical constants calculated from first principles.

After fitting the critical point using Eq. 4, the energy band structure calculated using first principles was used to plot the energy difference between the valence and conduction bands in the Brillouin zone, E_{cv}, and then the energy axis of the curve fitted to the critical point was aligned with the E_{cv} energy axis. It is worth noting that the bandgap calculated by the first nature principle is usually inconsistent with the experimental bandgap, so it is necessary to artificially shift E_{cv}, i.e., the bandgap is aligned, and the experimental bandgap of 0.25 eV[33] is used for Ta_2NiS_5 in this experiment. Then isoenergy lines are drawn from the center energy of the critical point to find the position tangent to E_{cv}, i.e., the position where the optical transition occurs [4]:

$$J_{cv}(E) = \frac{2}{(2\pi)^3} \int_{E_{cv}=E} \frac{dS}{\nabla_k (E_{cv})} \qquad \text{(S13)}$$

where J_{cv} is the joint density of states involved in the interband transition, S is the constant energy surface defined by $E_{cv} = E$, and $\nabla_k(E_{cv})$ is the gradient in the k-space. Obviously, with $\nabla_k(E_{cv}) = 0$ at the tangent point, the joint state density is in a singular state and undergoes an optical transition. Then the valence and conduction bands involved in the transition are obtained by E_{cv} and labeled in the energy band structure diagram, and then the atomic orbitals and carrier types involved in the transition are identified in combination with PDOS.

Table S2. Best-fit parameters for Ta2NiS5 critical point analysis.

$d^2\varepsilon_i/dE^2$ (i = a, b, c)	Center Energy(E0) [eV]	Phase Angle(ϕ) [°]	Amplitude(A) [F/m]	Broadening(Γ) [eV]
	0.53	133.25	2.04	0.10
	1.25	166.09	0.71	0.17
	1.53	200.40	1.25	0.10
	1.55	216.54	3.44	0.17
$d^2\varepsilon_a/dE^2$	2.38	200.23	0.04	0.07
	2.40	128.79	2.49	0.29
	2.97	173.25	0.05	0.12
	4.05	147.96	2.87	0.57
	5.28	134.71	3.40	0.78
	0.77	153.69	0.43	0.21
	1.61	78.70	0.01	0.03
	1.78	185.61	2.05	0.75
	1.85	145.67	2.98	0.42
$d^2\varepsilon_b/dE^2$	2.40	202.08	0.61	0.26
	3.15	145.67	1.72	0.58
	4.44	178.39	0.60	0.30
	5.29	154.18	0.88	0.60
	6.04	142.21	1.38	0.85
	0.75	197.42	2.72	0.21
	1.08	209.76	1.12	0.29
	1.59	162.26	3.84	0.14
	1.61	128.28	2.53	0.11
	1.76	128.25	2.94	0.14
$d^2\varepsilon_c/dE^2$	2.04	141.61	1.66	0.15
	2.32	160.06	1.64	0.26
	3.49	160.34	5.54	0.81
	3.80	190.11	0.85	0.39
	4.32	247.35	1.83	0.39
	5.74	169.58	0.97	0.92
